# Plasma and urinary exposure of cannabidiol and cannabidiolic acid in horses following consumption of contaminated feed

**DOI:** 10.1186/s13620-026-00350-6

**Published:** 2026-06-04

**Authors:** Carl Ekstrand, Peter Michanek, Elin Hernlund, Ronette Gehring, Matilda Salomonsson

**Affiliations:** 1 Department of Animal Biosciences, Swedish University of Agricultural Sciences, Uppsala, Sweden; 2 Department of Population Health Sciences, Division of Veterinary and Comparative Pharmacology, Utrecht University, Utrecht, the Netherlands; 3 Department of Chemistry, Environment and Feed Hygiene, Swedish Veterinary Agency, Uppsala, Sweden

**Keywords:** Equine, Cannabinoid exposure, Feed contamination, Inter-individual variability, Doping control, Inadvertent exposure

## Abstract

**Background:**

Cannabidiol (CBD) and cannabidiolic acid (CBDA) are non-psychoactive cannabinoids naturally present in hemp-derived products. While these compounds are prohibited during equine competition, horses may be inadvertently exposed through contaminated feed. Data describing systemic and urinary exposure to CBD and CBDA following such low-dose, involuntary intake are limited. This study aimed to describe plasma and urine concentration-time profiles of CBD and CBDA in horses after consumption of experimentally contaminated feed.

**Results:**

Twelve Standardbred horses received feed containing low doses (10–15 mg) of either CBD or CBDA twice daily for three days. Neither compound was detected in pre-administration samples. Following exposure, CBD was only sporadically quantifiable in plasma, whereas CBDA was detected in plasma from most horses for up to 48 h. In urine, both cannabinoids were quantifiable in all horses, with CBDA consistently present at higher concentrations and for longer durations than CBD. Maximum observed urinary concentrations were markedly higher for CBDA than for CBD. Considerable inter-individual variability was observed in both plasma and urine concentrations. Neither CBD nor CBDA was detected in plasma or urine for more than four days. Overall, systemic exposure following feed contamination was low compared with doses reported to produce therapeutic effects in horses or other species.

**Conclusions:**

Low-dose oral exposure to CBD and CBDA, mimicking feed contamination, can result in detectable concentrations of cannabinoids in equine plasma and urine, with CBDA representing the predominant analyte. Although the study was not designed to establish detection times and analytical sensitivity may differ from routine doping control, the findings demonstrate that inadvertent exposure through feed may lead to measurable cannabinoid concentrations despite doses well below those associated with reported pharmacological effects. These results highlight the importance of awareness of potential contamination sources and cautious interpretation of low-level cannabinoid findings in equine biological samples.

## Introduction

Cannabinoids are a group of substances naturally occurring in plants of the *Cannabis* species. The first isolated cannabinoid, cannabinol, was discovered in the 19th century, and its structure was elucidated in the 1930s [1]. To date, several other cannabinoids have been identified; however, cannabidiol (CBD), cannabidiolic acid (CBDA), and Δ9-tetrahydrocannabinol (THC) are those that have attracted the most interest in veterinary medicine due to their proposed clinical potential, such as analgesic, anti-inflammatory, and muscle-relaxant properties [2–4].

Regulatory bodies within racing and equestrian sports have rules against doping and medication during competition to protect horse welfare, preserve the integrity of the sport and ensure fair play [5, 6]. Refined analytical methods have continuously increased the sensitivity of plasma and urine analyses in racehorses. Consequently, even low concentrations of substances permitted for use outside competition, such as CBD and CBDA, may be detected in doping control samples. Moreover, CBD and CBDA have been reported at concentrations sufficiently high to cause an adverse analytical finding when hemp straw is used as bedding material for horses [7]. While the risks associated with bedding have been documented, the exposure from contaminated feed remains less well understood and warrants further investigation. This study aimed to describe the plasma and urine concentration-time courses of CBD and CBDA following consumption of experimentally contaminated feed.

## Methods

### Animals and preparation

Twelve Standardbreds, eight mares and four geldings were used in the experiment. The median (range) age was 14 years (3–21 years), and the median (range) body weight was 536 kg (468–676 kg). All horses were considered clinically free from diseases. The horses were housed in individual boxes during sampling periods. During the day, the horses were kept in paddocks or on pasture and returned to their boxes in the afternoon. The horses were fed haylage and concentrate consistent with everyday feeding routines throughout the experiment. Water was available *ad libitum* throughout the study. Before the last drug administration, the hair over one jugular vein was clipped, and the skin was pre-treated with a prilocaine–lidocaine cream (EMLA, 25 mg/g + 25 mg/g, Aspen Nordic, Ballerup, Denmark) prior to aseptically placement of a catheter (MILA, 14G [2.1 × 130 mm], Florence, Kentucky, USA) in the jugular vein.

### Drugs and chemicals

A commercially available CBD oil (Bedrolite 100 mg/mL, Cannabiszorg, Breukelen, the Netherlands) was used in the experiments. According to the manufacturer, the CBD preparation was produced in accordance with good manufacturing practice but contained traces of other cannabinoids (CBDA 0.16%, THC 0.45% and cannabichromene 0.45%). The CBDA (100 mg/mL) preparation in almond oil was compounded by Clinical Cannabis Care (Breukelen, The Netherlands) using CBDA raw material supplied by Purisys (Athens, GA, U.S.A).

The reference material CBD was purchased from Chiron AS (Trondheim, Norway), while CBDA and the internal standards CBD-d9 and CBDA-d3 were purchased from Cayman Chemical (Ann Arbour, USA). Water was purified using a Milli-Q purification system from Merck Life Science AB (Merck KGaA, Darmstadt, Germany). All other chemicals used were of analytical grade or higher.

### Experimental design

The trial was a two-treatment, randomised, crossover design with a minimum washout period of one week between treatments. Either CBD or CBDA oil was mixed with soaked sugar beet pulp (Betfor^®^, Nordic Sugar, Sweden) and administered individually to the horses twice daily (08:00 and 16:00) for three consecutive days. Administration was performed in separate feed buckets, which were visually inspected after feeding to confirm complete consumption. The CBD and CBDA dose was 15 mg per meal. Three horses (#4, #5 and #6) were unintentionally administered 10 mg CBD twice daily instead of 15 mg. Horse demographics and individual doses are presented in Table 1.

**Table 1:** Demographic and dose information from 12 standardbred horses given feed contaminated with cannabidiol (CBD) and cannabidiolic acid (CBDA) twice daily for three days

Horse	Age	Sex	Weight (kg)	Dose CBD/CBDA (mg)	Dose CBD/CBDA per kg (mg/kg)
1	3	M	515	15/15	0.029/0.029
2	21	M	468	15/15	0.032/0.032
3	13	M	562	15/15	0.027/0.027
4	10	F	530	10/15	0.019/0.028
5	10	F	502	10/15	0.020/0.030
6	16	M	528	10/15	0.019/0.028
7	12	F	573	15/15	0.026/0.026
8	15	F	542	15/15	0.028/0.028
9	21	F	575	15/15	0.026/0.026
10	20	F	479	15/15	0.031/0.031
11	14	F	602	15/15	0.025/0.025
12	14	F	676	15/15	0.022/0.022

Blood samples were collected before the first cannabinoid administration, immediately before the last cannabinoid administration (0 h), and at 1, 2, 3, 4, 5, 6, 16, 24, 48, 96 and 168 h after the last administration. During the first 24 h, blood was drawn through the indwelling catheter; the catheter was subsequently flushed with an isotonic saline solution equal to the withdrawn volume. The remaining samples were collected by venipuncture. Collected blood was dispensed into 2 K EDTA-coated tubes, immediately placed on ice, and centrifuged at 4 °C and 1800 × g for 10 min within one hour of collection. The supernatant was transferred to new tubes and stored at − 80 °C pending analysis.

Urine samples were collected before CBD/CBDA administration, at 0 h (before the last CBD/CBDA administration), and at 4, 16, 24, 48, 96, and 168 h after administration.Urine from geldings was collected using disposable plastic bags placed in standard urine collectors. In mares, urine samples were collected during spontaneous urination whenever possible; otherwise, urinary catheterisation was performed if spontaneous urination did not occur within a reasonable time period. Urine samples were stored at − 20 °C pending analysis.

The experimental protocol was approved by the Uppsala Animal Ethics Committee (5.8.18–02809/2020).

### CBD and CBDA analyses

The analytical method has previously been described by Ekstrand et al. [8] In brief, plasma samples were prepared by protein precipitation using acetonitrile containing internal standard prior to UHPLC-MS/MS analysis. Urine samples underwent enzymatic hydrolysis using β-glucuronidase and protease before solid phase extraction (SPE) and UHPLC-MS/MS analysis. CBD-d9 and CBDA-d3 were used as internal standards. An Acquity Ultrahigh Performance Liquid Chromatography (UPLC) coupled to a Xevo TQ-Sµ tandem quadrupole mass spectrometer (Waters Corporation, MA, USA) with an electrospray interface was used for the quantification of CBD and CBDA. For quantitative analysis, calibration curves consisting of eight calibrators were used (0.076–20.4 ng/mL CBD in plasma, 0.750–150 ng/mL CBDA in plasma, 0.482–48.2 ng/mL CBD in urine, low range 4.45–49.4 ng/mL CBDA in urine, and high range 39.5–988 ng/mL CBDA in urine). Calibration curves were constructed using the peak area ratio (CBD or CBDA/internal standard) as a function of CBD or CBDA concentration. Calibration functions were calculated by linear regression using a weighting factor of 1/x² for CBD and 1/x for CBDA. The results of the method validation (linearity, intra- and inter-day precision, and accuracy) were all within the acceptance criteria specified in the European Medicines Agency (EMA) guideline for bioanalytical method validation. Linearity was evaluated, and the R² values obtained for both compounds were > 0.99 on all days. In plasma, intra- and inter-day precision (RSD%) for CBD ranged from 1.8% to 12%, and for CBDA from 1.8% to 13%. In urine, precision ranged from 2.2% to 15% for CBD (9.4–19% at the LOQ) and from 0.4% to 3.1% for CBDA. In plasma, accuracy for CBD ranged from 100% to 110% (100–119% at the LOQ), while accuracy for CBDA ranged from 87% to 115%. In urine, accuracy for CBD ranged from 86% to 111%, and for CBDA from 96% to 104% (113–117% at the LOQ). The limit of quantification for CBD was 0.1 ng/mL in plasma and 0.5 ng/mL in urine, and for CBDA, 1.0 ng/mL in plasma and 5 ng/mL in urine.

## Results

### Cannabidiol and cannabidiolic acid in plasma

**Fig. 1:**
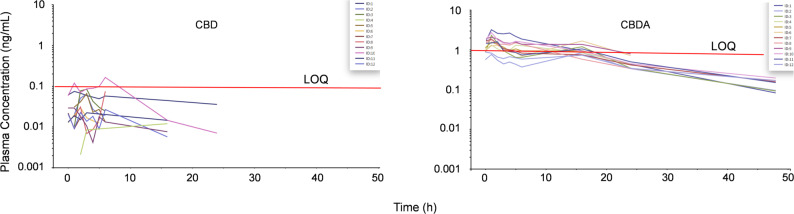
Spaghetti plot showing observed plasma cannabidiol (CBD) and cannabidiolic acid (CBDA) concentration–time profiles following oral administration in feed twice daily for three days, with the last administration at 0 h. Red horizontal lines indicate the lower limit of quantification (LOQ) for CBD (0.1 ng/mL) and CBDA (1 ng/mL), respectively. Please note that observations below the LOQ were approximated and should be interpreted with caution

Neither CBD nor CBDA were detected in any pre-administration samples. After the last administration, CBD could be detected in plasma for up to 24 h; however, plasma CBD concentrations were only quantified above the lower limit of quantification (LOQ, 0.1 ng/mL) on two occasions, measuring 0.12 ng/mL and 0.16 ng/mL, respectively (Fig. 1). All other plasma CBD concentrations were below the LOQ.

Plasma CBDA concentrations were detected in samples from all horses and were quantifiable (LOQ 1 ng/mL) in plasma from 10 horses (Fig. 1). Plasma CBDA could be detected for up to 48 h after the last administration. The maximum observed plasma CBDA concentration had a median (range) of 1.85 ng/mL (1.3–3.2 ng/mL). Neither CBD nor CBDA were detected in plasma samples collected 72–96 h after the last administration.

### Cannabidiol and cannabidiolic acid in urine

Neither CBD nor CBDA were detected in any pre-administration urine samples. Urine CBD concentrations were quantifiable (LOQ 0.5 ng/mL) in samples from all horses. Cannabidiol could be detected in urine for up to 96 h after the last CBD administration, but only in one horse. In the remaining horses, CBD was quantified in urine for up to 24 h after the last CBD administration. The maximum observed urine CBD concentration had a median (range) of 5.6 ng/mL (2.6–23.9 ng/mL).

**Fig. 2:**
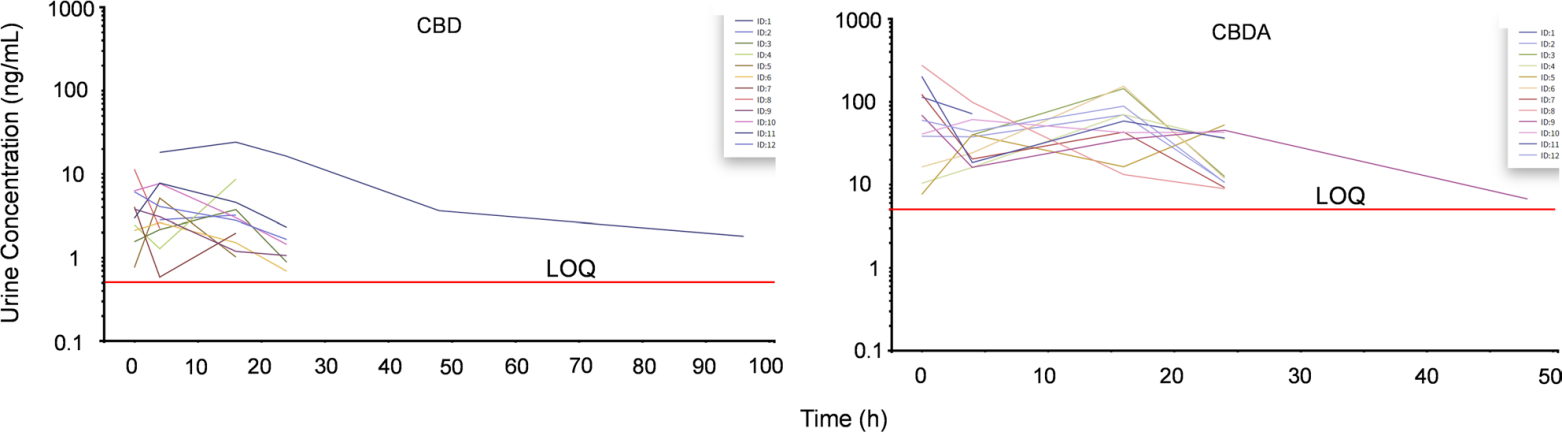
Spaghetti plot showing observed urine cannabidiol (CBD) and cannabidiolic acid (CBDA) concentration–time profiles following oral administration in feed twice daily for three days, with the last administration at 0 h. Red horizontal lines indicate the lower limit of quantification (LOQ) for CBD (0.5 ng/mL) and CBDA (5 ng/mL), respectively

Urine CBDA concentrations were quantifiable (LOQ 5 ng/mL) in samples from all horses. The maximum observed urine CBDA concentration had a median (range) of 100.4 ng/mL (38.1–273.0 ng/mL). Neither CBD nor CBDA were detected in urine samples collected 168 h after the last administration. The urine CBD and CBDA concentration–time courses are shown in Fig. 2.

## Discussion

Doping and medication control for horses participating in racing or equestrian sports is important for several reasons. Protecting horse welfare is one reason, because analgesic or anti-inflammatory agents can mask injuries and increase the risk of injury to horses. To safeguard fair play and the integrity of the sport are other reasons, as the use of prohibited substances and the misuse of controlled medications undermine equal competition. Both objectives ultimately rely on robust scientific data describing exposure, elimination and variability of prohibited or controlled substances in horses.

The control systems must also be reliable. In contrast to deliberate administration, involuntary exposure through feed or bedding is difficult to monitor and control at the individual horse level, increasing the risk of unintended adverse analytical findings. The analytical methods today are sensitive, and small amounts of substances can be detected in doping control. To avoid adverse analytical findings resulting from inadvertent intake of CBD and/or CBDA, trainers, veterinary surgeons, and feed producers need data to make well-informed decisions. Today, such data are sparse in peer-reviewed literature. In an experimental study on three horses, the horses were positive for cannabinoids for up to 48 h when hemp straw was used as bedding material [7]. However, that study included only urine analyses and a limited number of animals. In our recent publication investigating plasma exposure following oral administration of CBD and CBDA in horses [8], the primary focus was comparative systemic exposure after controlled administration of substantially higher cannabinoid doses. In contrast, the present study was specifically designed to investigate plasma and urinary exposure following repeated low-dose administration intended to mimic inadvertent exposure through contaminated feed. Consequently, the present study provides novel information regarding prolonged low-level exposure, urinary elimination, inter-individual variability, and potential implications for equine doping control under realistic contamination scenarios. Consistent with previous findings, the present study demonstrates that CBD could be detected for at least 24 h and 96 h in plasma and urine, respectively, with marked inter-individual variability. Cannabidiolic acid was detectable in plasma and urine for at least 48 h. The plasma and urine CBDA concentrations in the current study were consistently higher than corresponding CBD concentrations. This was consistent with previous publications [9–11] and further supports the notion that systemic exposure after oral administration is greater for CBDA than for CBD in horses. This difference may reflect differences in oral bioavailability, elimination or first-pass metabolism between CBDA and CBD, although the exact mechanisms remain to be elucidated.

There is limited data on the response of horses to cannabinoids. Some studies have reported on decreased lameness and improved quality of life [3, 4], and there are also case reports on other conditions, such as stereotypic behaviour and mechanical allodynia [12, 13]. Unfortunately, all those studies were qualitative in assessing the cannabinoid response, and quantitative data, such as potency values for horses, remain to be estimated. As a consequence, it is currently not possible to relate measured plasma or urine concentrations of cannabinoids to defined pharmacodynamic effects in horses. However, the oral doses (approximately 250 mg for a 500 kg horse) used in studies demonstrating any clinical efficacy [3, 4, 12, 13] were approximately 20-fold higher than those in the current study (10–15 mg per horse). Moreover, an oral dose of 2 mg/kg twice daily has been shown to be effective in osteoarthritic dogs [14], and doses as high as 75 mg/kg have been estimated to be effective in 50% of treated mice [15]. In comparison, the doses used in the current study were low, and it could be argued that the resulting plasma exposure would be insignificant for a therapeutic effect. This observation highlights the important distinction between pharmacological relevance and analytical detectability in the context of doping control, as previously described [5, 16]. Another important aspect in doping control is the use of screening limits (SLs), which aim to avoid adverse analytical findings caused by trace concentrations without pharmacological relevance or by inadvertent contamination [17]. The present study provides valuable information regarding plasma and urinary cannabinoid concentrations following exposure to contaminated feed, which may contribute to future discussions on appropriate SLs for CBD and CBDA. However, establishment of SLs would require knowledge of clinically effective plasma concentrations, which are currently lacking for both compounds in horses.

Of additional relevance to doping control is the extensive metabolism of CBD to metabolites such as 7-carboxy-cannabidiol (COOH-CBD) and 7-hydroxy-cannabidiol (OH-CBD) (18, 19). This extensive oxidative metabolism may partly explain the comparatively low plasma concentrations of CBD observed in the present study. Interestingly, the difference between CBDA and CBD concentrations was substantially greater in plasma than in urine. Since all urine samples were hydrolysed prior to analysis, conjugated metabolites would partly have been deconjugated before quantification, potentially increasing measured urinary parent CBD concentrations. However, oxidized metabolites such as OH-CBD and COOH-CBD were not quantified in the present study, and their contribution to the observed differences between CBD and CBDA therefore remains unclear.

It should be noted that (i) this was not a detection time study and the analytical methods used in doping control could be more or less sensitive than in the current study, and (ii) there is a marked variability between the 12 horses included in this study. Consequently, it is reasonable to believe that CBD/CBDA could be detected for an extended time in some individuals using a larger population of horses. This variability implies that a small proportion of horses may be at higher risk of prolonged detectability following similar low-dose exposures. Hence, if exposure to contaminated feed is suspected or confirmed, an appropriate stand-down period before competition may be necessary to reduce the risk of adverse analytical findings [18]. Furthermore, it has been shown that the CBD terminal half-life after repeated oral administration was 161 h compared with 10 h after a single oral dose [19, 20]. Therefore, CBD could possibly be detected for an extended time in horses repeatedly exposed to low doses of CBD and/or CBDA in feed for longer periods than the three days that were used in the present study. Taken together, these findings underline the need for cautious interpretation of low-level cannabinoid findings in doping control samples and for increased awareness of inadvertent exposure routes among veterinarians and horse caretakers.

## Conclusions

This study demonstrates that low-dose oral exposure to cannabidiol and cannabidiolic acid, mimicking feed contamination, can result in measurable concentrations of cannabinoids in both plasma and urine of horses, with considerable inter-individual variability. Cannabidiolic acid consistently showed higher systemic and urinary exposure than cannabidiol, supporting previous observations that CBDA may be the predominant compound detected following oral intake of hemp-derived products in horses. The study was not designed to determine detection times, and the analytical sensitivity may differ from that used in routine doping control. In addition, the limited number of horses and the short exposure period restrict extrapolation to prolonged or repeated low-level exposures. Nevertheless, the findings highlight that inadvertent exposure to cannabinoids through contaminated feed may lead to detectable concentrations in biological samples, despite doses well below those associated with reported therapeutic effects. These results underline the importance of awareness of potential contamination sources and of cautious interpretation of low-level cannabinoid findings in equine doping control.

## Data Availability

The datasets used and/or analysed during the current study are available from the corresponding author on reasonable request.

## References

[1] Mechoulam R, Hanuš LO, Pertwee R, Howlett AC. Early phytocannabinoid chemistry to endocannabinoids and beyond. Nat Rev Neurosci. 2014;15(11):757–64. 10.1038/nrn3811. 25315390 10.1038/nrn3811

[2] De Briyne N, Holmes D, Sandler I, Stiles E, Szymanski D, Moody S, et al. Cannabis, cannabidiol oils and tetrahydrocannabinol—what do veterinarians need to know? Anim (Basel). 2021;11(3). 10.3390/ani11030892. 10.3390/ani11030892 PMC8003882 33804793

[3] Aragona F, Tabbì M, Gugliandolo E, Giannetto C, D’Angelo F, Fazio F, et al. Role of cannabidiolic acid or the combination of cannabigerol/cannabidiol in pain modulation and welfare improvement in horses with chronic osteoarthritis. Front Vet Sci. 2024;11:1496473. 10.3389/fvets.2024.1496473. 39720409 10.3389/fvets.2024.1496473 PMC11668182

[4] Interlandi C, Tabbì M, Di Pietro S, D’Angelo F, Costa GL, Arfuso F, et al. Improved quality of life and pain relief in mature horses with osteoarthritis after oral transmucosal cannabidiol oil administration as part of an analgesic regimen. Front Vet Sci. 2024;11:1341396. 10.3389/fvets.2024.1341396. 38379920 10.3389/fvets.2024.1341396 PMC10876772

[5] Toutain PL. Veterinary medicines and competition animals: the question of medication versus doping control. Handb Exp Pharmacol. 2010;199315–39. 10.1007/978-3-642-10324-7_13. 10.1007/978-3-642-10324-7_13 20204593

[6] Wong JK, Wan TS. Doping control analyses in horseracing: a clinician’s guide. Vet J. 2014;200(1):8–16. 10.1016/j.tvjl.2014.01.006. 24485918 10.1016/j.tvjl.2014.01.006

[7] Trevisiol S, Popot MA, Garcia P, Boyer S, Caroff M, Drif L, et al. In vivo comparative study of hemp straw exposure and cannabidiol oil administration in horse urine. Drug Test Anal. 2024. 10.1002/dta.3783. 39118356 10.1002/dta.3783

[8] Ekstrand C, Michanek P, Hernlund E, Gehring R, Spjut K, Salomonsson M. Differences in plasma exposure of cannabidiol and cannabidiolic acid following oral administration to horses. J Vet Pharmacol Ther. 2026;49(1):22–32. 10.1111/jvp.70027. 41017237 10.1111/jvp.70027 PMC12796783

[9] Thomson ACS, McCarrel TM, Zakharov A, Gomez B, Lyubimov A, Schwark WS, et al. Pharmacokinetics and tolerability of single-dose enteral cannabidiol and cannabidiolic acid rich hemp in horses (Equus caballus). Front Vet Sci. 2024;11:1356463. 10.3389/fvets.2024.1356463. 38681854 10.3389/fvets.2024.1356463 PMC11047043

[10] Wang TC, Wakshlag JJ, Jager MC, Schwark WS, Trottier NL, Chevalier JM, et al. Chronic oral dosing of cannabidiol and cannabidiolic acid full-spectrum hemp oil extracts has no adverse effects in horses: a pharmacokinetic and safety study. Am J Vet Res. 2025;1–10. 10.2460/ajvr.24.08.0235. 10.2460/ajvr.24.08.0235 39787699

[11] Sosa-Higareda M, Guzman DS, Knych H, Lyubimov A, Zakharov A, Gomez B, et al. Twice-daily oral administration of a cannabidiol and cannabidiolic acid-rich hemp extract was well tolerated in orange-winged Amazon parrots (Amazona amazonica) and has a favorable pharmacokinetic profile. Am J Vet Res. 2023;84(4). 10.2460/ajvr.22.11.0197. 10.2460/ajvr.22.11.0197 36795552

[12] Cunha RZ, Felisardo LL, Salamanca G, Marchioni GG, Neto OI, Chiocchetti R. The use of cannabidiol as a novel treatment for oral stereotypic behaviour (crib-biting) in a horse. Vet Anim Sci. 2023;19:100289. 10.1016/j.vas.2023.100289. 36824298 10.1016/j.vas.2023.100289 PMC9941357

[13] Ellis KL, Contino EK. Treatment using cannabidiol in a horse with mechanical allodynia. Equine Vet Educ. 2021;33(4):e79–82. 10.1111/eve.13168.

[14] Gamble LJ, Boesch JM, Frye CW, Schwark WS, Mann S, Wolfe L, et al. Pharmacokinetics, safety, and clinical efficacy of cannabidiol treatment in osteoarthritic dogs. Front Vet Sci. 2018;5:165. 10.3389/fvets.2018.00165. 30083539 10.3389/fvets.2018.00165 PMC6065210

[15] McDonald JD, Zhou F, Kulpa J, Kuehl PJ. Preclinical assessment of pharmacokinetics and anticonvulsant activity of CBDTech, a novel orally administered cannabidiol (CBD) formulation for seizure and epilepsy. J Cannabis Res. 2025;7(1):73. 10.1186/s42238-025-00322-7. 41029806 10.1186/s42238-025-00322-7 PMC12487286

[16] Toutain PL. Veterinary medicines and competition animals: the question of medication versus doping control. Handb Exp Pharmacol. 2010:(199):315–39. 10.1007/978-3-642-10324-7_1m. 20204593 10.1007/978-3-642-10324-7_13

[17] Toutain PL, Lassourd V. Pharmacokinetic/pharmacodynamic approach to assess irrelevant plasma or urine drug concentrations in postcompetition samples for drug control in the horse. Equine Vet J. 2002;34(3):242–918. 12108741 10.2746/042516402776185985

[18] Toutain PL. How to extrapolate a withdrawal time from an EHSLC published detection time: a Monte Carlo simulation appraisal. Equine Vet J. 2010;42(3):248–54. 10.1111/j.2042-3306.2010.00028.x. 20486982 10.1111/j.2042-3306.2010.00028.x

[19] Eichler F, Poźniak B, Machnik M, Schenk I, Wingender A, Baudisch N, et al. Pharmacokinetic modelling of orally administered cannabidiol and implications for medication control in horses. Front Vet Sci. 2023;10:1234551. 10.3389/fvets.2023.1234551. 37621871 10.3389/fvets.2023.1234551 PMC10445762

[20] Ryan D, McKemie DS, Kass PH, Puschner B, Knych HK. Pharmacokinetics and effects on arachidonic acid metabolism of low doses of cannabidiol following oral administration to horses. Drug Test Anal. 2021;13(7):1305–17. 10.1002/dta.3028. 33723919 10.1002/dta.3028

